# The impact of arachnoid structures on skull-base meningioma surgical management: a radiological analysis and narrative review

**DOI:** 10.25122/jml-2024-0349

**Published:** 2024-07

**Authors:** Gheorghe Ungureanu, Alexandru Florian, Stefan Ioan Florian

**Affiliations:** 1 Iuliu Hatieganu University of Medicine and Pharmacy, Cluj-Napoca, Romania; 2 Neurosurgery Department, Cluj County Emergency Hospital, Cluj-Napoca, Romania

**Keywords:** meningioma, meningeal neoplasms, skull base neoplasms, arachnoid, subarachnoid space

## Abstract

The presence of intact arachnoid membranes between skull base meningiomas and critical neurovascular structures is crucial for predicting surgical outcomes, understanding tumor development and growth, and planning the feasibility of tumor resection or the need for adjuvant treatments. While neurosurgeons often utilize the subarachnoid cisterns to enhance access to these tumors and facilitate their removal, a comprehensive review aimed at health professionals involved in the diagnosis and treatment of this complex pathology, including radiologists, neurologists, oncologists, ophthalmologists, and neurosurgeons is still lacking. This study aims to summarize the interaction between skull base meningiomas, subarachnoid cisterns, and arachnoid membranes, emphasizing their significance in both the diagnosis and treatment of this pathology. By conducting a thorough radiological assessment of skull base meningiomas, correlating these findings with intraoperative observations, and reviewing relevant literature, we summarize the critical relationship between skull base meningiomas and the surrounding subarachnoid spaces. We concisely describe how arachnoid structures influence tumor growth and interaction with neurovascular elements. We advocate for the inclusion of tumor-arachnoid relationships in the medical literature concerning the treatment of these tumors. A better understanding and description of the interaction between tumors and neurovascular structures will aid in planning and attempting safer treatments, minimizing surgical risks, predicting potential tumor progression, and the need for adjuvant treatments.

## INTRODUCTION

The presence of intact arachnoid membranes between skull base meningiomas and critical neurovascular structures plays a pivotal role in predicting the feasibility of total tumor resection [1,2]. Neurosurgeons utilize the subarachnoid cisterns to enhance access to these tumors and facilitate their removal. In neurosurgical practice, arachnoid structures are widely acknowledged for their importance, yet a comprehensive understanding of their implications remains incomplete [3]. A major challenge stems from using common terminology with inconsistent and ambiguous criteria, which often results in grouping tumors with markedly different surgical characteristics into the same categories [4–7]. Nonetheless, in the various research articles proposing criteria for classifying skull-base meningiomas, authors have examined the relationship between tumors and the surrounding anatomical structures, with particular emphasis on the role of arachnoid membranes in shaping these interactions. This article aims to address this gap by investigating the interactions between skull base meningiomas, subarachnoid cis-terns, and arachnoid membranes, highlighting their relevance for neurosurgical practice. Through a detailed review of existing research, combined with an in-depth radiological analysis of skull-base meningiomas, supported by intraoperative findings from a personal series of patients operated for skull-base meningiomas, we propose a surgical concept that emphasizes the crucial role of arachnoid structures. Furthermore, we explore how these membranes may affect tumor growth and interaction with neuro-vascular elements, advocating for their inclusion in future classification systems. By understanding these relationships, neurosurgeons can better plan surgeries, attempt safer resections, minimize surgical risks, and predict potential tumor progression.

## MATERIAL AND METHODS

### Retrospective analysis of patient data

A retrospective analysis was conducted on the radiological data of patients who underwent craniotomy for the resection of skull base meningiomas, performed by the senior author (ISF) at our institution between 2015 and 2020. Tumor origin was determined using MRI Constructive Interference in Steady State (CISS) when available or T2 sequences corroborated with intraoperative notes. All the images were reviewed by two of the authors (GU and IAF). We excluded giant tumors (over 6 cm), those extending across all cranial fossae, and those patients who did not have adequate MRI imaging (either CISS or 3T thin-slice T2 MRI). We sought to identify patterns of tumor extension by comparing tumors located in similar regions and examining the relationship between the meningiomas, the overlying arachnoid membranes, and regional neurovascular structures. The presence of an arachnoidal cleavage plane, delineating the tumors from adjacent normal anatomical structures, was assessed intraoperatively and described in all cases. The preoperative radiological findings were systematically correlated with intraoperative observations.

### Literature review

A comprehensive literature review was performed using the Medline and EMBASE databases. The search strategy incorporated the following medical subject heading (MeSH) terms: 'classification’ and 'meningioma’, combined with 'suprasellar’, 'tuberculum’, 'planum sphenoidale’, 'clival’, “'petroclival’, and 'cerebellopontine’. Studies were classified based on the anatomical region of interest. Key anatomical factors considered important in the surgical management of skull base meningiomas were extracted and analyzed. Particular attention was given to how previous studies described tumor interactions with surrounding neurovascular structures and the role of arachnoid membranes in influencing surgical strategies. Our findings were then compared with the existing literature to synthesize the role of arachnoid structures in tumor progression and surgical resection..

## RESULTS

### Case summary

A total of 169 skull base meningiomas treated over the specified period were initially reviewed. After excluding those tumors for which imaging criteria did not meet our inclusion criteria, we were left with 110 cases. A summarized description of these cases is presented in Table 1.

**Table 1:** Tumor location, gender, and age of the patients

Location	Women	Men	Age (SD)
Suprasellar Tuberculum sellae Planum sphenoidale Clinoidal	40 24 **3** 13	22 12 3 7	55.5 (8)
Sphenoid wing	6	6	58.3 (9.7)
Clival	11	10	58.4 (1)
Cerebellopontine angle	11	4	62.7 (6.5)

**Figure 1:**
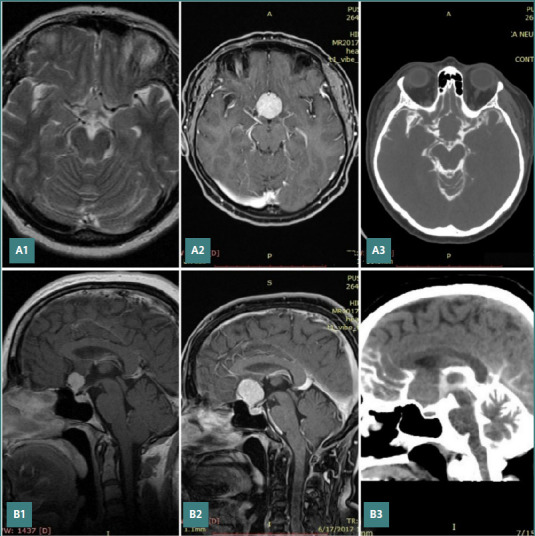
MRI and CT cisternography images depicting the relation between a meningioma arising in the suprasellar region and the subarachnoid cisterns and how tumor growth displaces the arachnoid structures. The patient (a 53-year-old woman) first presented to our service in 2013 with headaches and underwent an MRI. A1, B1, the initial MRI showed a small meningioma originating at the level of the tuberculum sellae. She refused any surgical intervention. In 2017, she presented with visual disturbances. A2, B2, the MRI reveals tumor growth. CT cisternography shows the preservation of the arachnoid layers and displacement of the cisterns, confirming the mentioned concept of tumor development. A3, B3, CT cisternography shows the preservation of the arachnoid layers and displacement of the cisterns, confirming the mentioned concept of tumor development.

The imaging study demonstrated that, despite variations in radiological characteristics such as contrast enhancement and size, the tumors displaced neurovascular structures in a predictable manner. MRI further confirmed that the tumors consistently displaced the arachnoid membranes in a reproducible pattern (Figure 1). These findings are consistent with previous reports [1]. The observed relationship between the tumors and the surrounding arachnoid elements allowed for reliable prediction of the tumors’ interactions with neurovascular structures within the affected subarachnoid cisterns based on imaging. These predictions were consistently validated by intraoperative observations.

Based on our findings, we developed models for each tumor type, incorporating the interaction with arachnoid structures to predict the involvement of various neurovascular structures by the tumor. We then assessed the consistency of our results with those reported by other researchers. A strong correlation was observed between our radiological and intraoperative findings and those documented in other studies. Key observations were synthesized for the supratentorial and infratentorial compartments, detailed below.

### Key findings for the supratentorial compartment

Several classification systems exist for supratentorial meningiomas, often utilizing similar terminology and anatomical landmarks to describe tumors originating in the suprasellar region. However, there is considerable variability in how these terms are applied across different classifications [2-5]. While there is some debate regarding naming various arachnoid cisterns in this area, most authors consistently recognize the chiasmatic, carotid, and lamina terminalis cisterns [6,7]. Notably, the chiasmatic cistern includes both prechiasmatic and postchiasmatic components [7].

Based on our analysis of radiological and intraoperative data, along with the comprehensive literature review, we reached the following conclusions:

### Classification challenges

Distinguishing tumors located in the planum sphenoidale, tuberculum sellae, or diaphragma sellae regions based solely on MRI appearance can be challenging and highly subjective, particularly as tumor size increases and spans multiple regions, obscuring the precise point of origin. However, our findings indicate that both contrast-enhanced MRI and CISS (Constructive Interference in Steady State) sequences offer valuable and reliable markers for this purpose. On contrast-enhanced MRI, the dura at the tumor’s site of origin exhibits greater contrast enhancement and thickening compared to regions affected at later stages. CISS sequences further aid differentiation by revealing a predictable invasion pattern of the arachnoid cisterns based on the tumor’s point of origin. These imaging techniques allow for an estimation of the tumor’s origin by analyzing the displacement of surrounding anatomical structures.

**Planum sphenoidale meningiomas:** Tumors originating from the planum sphenoidale typically extend anteriorly and superiorly into the prechiasmatic cistern, with posterior growth limited by the optic chiasm and lamina terminalis cisterns, leading to an anterior and upward trajectory. In the cases we operated on with this pathology, we consistently observed that multiple arachnoidal membranes covering critical structures such as the optic nerves, internal carotid artery (ICA), and pituitary stalk facilitated resection by providing a protective barrier. This allowed for total resection in all cases. Our findings align with those reported by other authors in their surgical series [8].

**Tuberculum sellae meningiomas:** These tumors involve both the prechiasmatic and retrochiasmatic compartments of the chiasmatic cistern, closely interacting with the optic chiasm. As they expand, they compromise the cistern’s integrity, extending superiorly and displacing the optic nerves and ICAs laterally while shifting the anterior cerebral arteries (ACAs) and the optic chiasm upwards. In all our patients with tuberculum sellae tumors, the membrane of Lilequist was intact, and removing the posterior part of the tumor was easily achieved. In no case did we notice a posterior growth towards the clivus. We also found that although these tumors may invade the optic canal, the optic nerves typically remain safeguarded by the arachnoid coverings originating in the prechiasmatic compartment, and total removal of the tumors is possible. An intact arachnoid membrane is also a strong predictor of favorable visual outcomes. We do not routinely perform the opening of the optic canal, although this is suggested by other researchers [9,10]. Involvement of the optic canal is generally well visualized on fine-section MRI and during surgery. Our literature review revealed several classification systems for these tumors [11,12]. We find that the Mortazavi scale is straightforward to apply, and given the need for a more standardized classification system, we recommend its use in studies focusing on the surgical treatment of tuberculum sellae meningiomas.

**Diaphragma sellae meningiomas:** Tumors in this region can extend either anteriorly or posteriorly relative to the chiasm. In the anterior spread, the arachnoid membranes are displaced posteriorly, separating the tumor from the pituitary stalk and chiasmatic cistern contents. Conversely, retrochiasmatic tumors push the arachnoid anteriorly, protecting visual elements but often leaving the pituitary stalk exposed. Our intraoperative findings, consistent with other studies, indicate that these tumors do not encase the stalk when anterior to the chiasm but do so when retrochiasmatic, affecting postoperative hormonal function [4,8].

**Anterior clinoid process meningiomas:** Meningiomas originating from the inferior aspect of the anterior clinoid process frequently envelop the internal carotid artery (ICA) directly, a phenomenon due to the artery traversing between two arachnoidal planes, as initially described by Al-Mefty [13]. Our intraoperative observations indicate that these tumors typically weaken the arterial wall, and tumor dissection from the arterial wall can result in intraoperative arterial rupture. In our series, patients with an intact arachnoid membrane protecting the artery did not exhibit vessel narrowing on preoperative MRI. In contrast, arterial injury occurred in three patients who showed 180° encasement of the carotid artery and narrowing evident on preoperative MRI. Based on these findings, we recommend taking additional precautions in cases where vessel encasement and narrowing are observed on preoperative imaging due to the increased risk of arterial injury. While vessel narrowing does not necessarily indicate arterial wall involvement, such involvement is more likely when the vessel diameter is reduced. Additionally, our findings indicate the presence of an arachnoid membrane covering the optic nerve on the tumor side in most cases, similar to what is observed in tuberculum sellae meningiomas. In our series, this feature was associated with favorable visual outcomes. Several other studies have also identified the presence of this membrane as a positive prognostic factor for postoperative visual improvement [14]. Several classification systems exist for anterior clinoid meningiomas, with the one developed by Al-Mefty *et al*. being the most widely recognized [15-20]. We recommend its use in relevant studies [15]. However, we believe that distinguishing between group I and II meningiomas based on preoperative MRI is rarely feasible, and therefore, this classification should be applied only after intraoperative evaluation.

### Key findings for the infratentorial compartment

**Figure 2:**
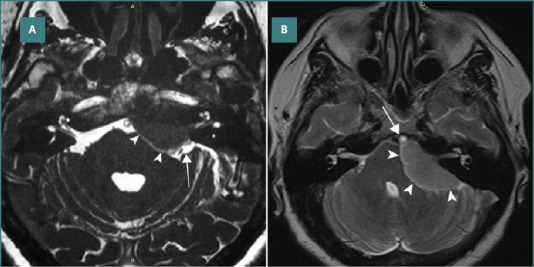
MRI images depict the relation between skull base meningiomas and the subarachnoid cisterns in the posterior fossa. A, shows a meningioma arising at the level of the prepontine cistern. The arachnoid membrane is pushed posteriorly (arrowheads). The cerebellopontine cistern and the neurovascular elements within are pushed laterally and inferiorly (arrow). B, shows a meningioma arising at the level of the cerebellopontine cistern. The arachnoid membrane is pushed medially (arrowheads), and no cleavage plane exists between a tumor and the neurovascular elements within this cistern. The prepontine cistern and the neurovascular elements within are pushed medially (arrow).

Due to the high density of neurovascular elements in the limited space of the infratentorial fossa, the significance of the arachnoid membranes for the safe resection of meningiomas, while preserving neurological functions, is even more critical than in the supratentorial region. Similar to the supratentorial region, various classification systems for posterior fossa meningiomas rely on approximately the same anatomical elements, though the limited space makes identifying these elements on MRI images a cumbersome task. In analyzing our case series of posterior fossa meningiomas, we found that more so than with supratentorial tumors, we had to use similar terminology to describe essentially different tumors that were, in fact, distinct in terms of their surgical prognosis. This discrepancy may contribute to the varying results of treatment strategies reported in the literature, as we use the same terms for different tumors, as noted by other authors [21]. Nonetheless, since meningiomas develop by distributing along the subarachnoid cisterns of the posterior fossa, incorporating the relationship between the tumor and arachnoid compartments into the existing nomenclature can provide a more nuanced understanding of these tumors and the expected surgical outcomes. The analysis of the interaction between the tumors and arachnoid membranes reveals the following:

**Midline tumors:** Tumors located at the level of the prepontine cistern typically displace the basilar artery and brainstem posteriorly and laterally (Figure 2A). Liliequist’s membrane often impedes their upward extension. When these tumors extend through the interpeduncular cistern, they may further expand into the supratentorial space. The anterior pontine membrane separates these tumors from the cerebellopontine cistern and the fifth, seventh, and eighth cranial nerves. Tumors originating outside the prepontine cistern displace the arachnoid membranes toward the brainstem and cranial nerves, facilitating total resection. Tumors originating in the prepontine cistern can be either 'true’ petroclival meningiomas or 'midclival’ meningiomas, as defined by Al-Mefty and colleagues [22]. In these cases, the fifth cranial nerve is displaced laterally, while the seventh and eighth cranial nerves are pushed laterally and inferiorly. In midclival tumors, the basilar artery is often encased by the tumor, and the cranial nerves are pushed bilaterally. When the arachnoid membranes of the prepontine and cerebellopontine cisterns remain intact, and the tumor is not excessively solid, total resection is generally achievable.

**Cerebellopontine cistern tumors:** Tumors located at the level of the cerebellopontine cistern displace the trigeminal nerve superiorly and posteriorly and the facial-vestibular bundle inferiorly and laterally (Figure 2B). Dissection of these tumors while preserving the function of the seventh and eighth nerves can be highly challenging. Tumor extension along the trajectory of the trigeminal nerve is possible. The lateral pontomedullary arachnoid membrane creates a cleavage plane between these tumors and the glossopharyngeal and vagus nerves.

**Figure 3:**
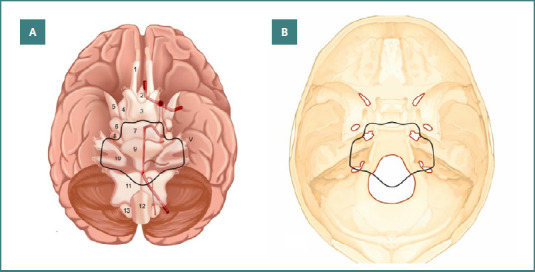
Illustration of the subarachnoid cisterns and their relationship with the primary neurovascular structures. A, the black diagram delineates the landmarks of a “high-risk” area where surgery carries a high risk. B, the same layout is represented at the level of a skull base. Legend: 1. Olfactory cistern; 2. Lamina terminals cistern; 3. Chiasmatic cistern; 4. Carotid cistern; 5. Sylvian cistern; 6. Crural cistern; 7. Interpeduncular cistern; 8. Ambient cistern; 9. Prepontine cistern; 10. Cerebellopontine cisterns; 11. Cerebellomedullary cisterns; 12. Anterior spinal cistern; 13. Posterior spinal cistern; V. Trigeminal nerve

**Premedullary cistern tumors:** Tumors arising at the level of the premedullary cistern develop anteromedially in ascending and descending directions, with their growth limited posteriorly by the dentate ligament. As they grow, these tumors displace the glossopharyngeal, vagal, and accessory nerves laterally. However, a cleavage plane can usually be identified as these nerves are contained within other cisterns.

**Cerebellomedullary cistern tumors:** Tumors at the level of the cerebellomedullary cistern displace the glossopharyngeal, vagal, and accessory nerves posteriorly. These tumors are more challenging to dissect from these nerves due to the absence of multiple arachnoid layers.

**Tumors on the petrous bone:** Tumors originating outside the cerebellopontine cistern on the petrous bone displace the elements contained within this cistern (the facial and vestibulocochlear nerves, and in larger tumors, also the glossopharyngeal and vagal nerves) towards the medial side. The displacement of the cisterns and the multiple arachnoid membranes usually creates an excellent cleavage plane between these tumors and the neurovascular elements, facilitating surgical resection.

**'High-risk’ area tumors:** Tumors originating in the area delineated by the posterior clinoid, trigeminal nerve, internal acoustic meatus, jugular foramen, and dentate ligament pose the highest surgical risk. Their excision is associated with a higher incidence of postoperative deficits, as also noted by other authors [23-25]. This 'high-risk’ area corresponds to the medial side of the adjacent arachnoid compartments, making the potential complications associated with tumor resection easily predictable by carefully analyzing the typical displacement of the subarachnoid cisterns on MRI CISS imaging, even when other anatomical elements are not easily recognized (Figure 3AB).

## DISCUSSION

Our study aims to provide a comprehensive overview of the interaction between arachnoid membranes and skull-base meningiomas and how these structures contribute to tumor resection. Despite the acknowledged importance of the arachnoid in the safe surgical treatment of skull-base meningiomas, to our knowledge, no previous study has summarized the significance of this interaction across the major categories of these tumors. This research highlights the complex relationship between meningiomas and arachnoid membranes, as well as their impact on adjacent neurovascular structures. By clarifying this interaction, we offer valuable insights for improving diagnostic accuracy and guiding treatment decisions. Additionally, we emphasize the critical role of subarachnoid cisterns in both tumor progression and surgical resection and review key findings from the literature regarding this relationship.

Understanding this connection can aid medical professionals in predicting the relationship between tumors and neurovascular structures, improving the diagnosis accuracy of these patients, assessing tumor resectability, comprehending the extension patterns of meningiomas, and planning appropriate treatment strategies. With cerebral imaging now routine, an increasing number of patients are presenting with small, asymptomatic lesions. Given that tumors originating from specific locations tend to follow consistent growth patterns, it is often possible to reliably predict tumor progression in cases of small tumors. This enables clinicians to offer more tailored treatment options for patients with small tumors, allowing for accurate prediction of which structures will be affected and how.

While the neurosurgical literature extensively emphasizes the importance of intact arachnoid membranes in separating tumors from neurovascular components to achieve successful surgical resection of skull base meningiomas without causing unacceptable morbidity, there is a paucity of studies addressing how the arachnoid-tumor relationship can be utilized in tumor diagnosis, treatment planning and in better informing patients regarding the risks associated with the surgical treatment. Analyzing MRI sequences is crucial for identifying the neurovascular elements encountered during surgery and their relationship with the tumor. New MRI techniques, such as super-selective Diffusion Tensor Tractography (DTT), can occasionally map the trajectory of cranial nerves adjacent to or traversing tumors. However, their application remains limited, as various factors can influence their effectiveness [26,27].

The use of the more common MRI CISS imaging to predict the encasement, isolation, or displacement of cranial nerves and cerebral arteries by the tumor allows us to understand how a tumor distorts arachnoid cisternal anatomy. At our institution, this technique is routinely employed in patients with skull base pathology, ventricular tumors, and suspected neurovascular conflicts (e.g., trigeminal neuralgia). While some studies have suggested that its use may increase costs, in our practice, this technique has been integrated into standard protocols without incurring additional expenses or significantly extending procedure time [28]. However, cost and time implications may vary between institutions and countries. Analyzing the interaction between the tumor and overlying arachnoid structures allows for accurate prediction of neurovascular involvement, even when these elements are poorly visualized compared to other conventional MRI techniques. This approach improves diagnostic precision, optimizes treatment planning, and facilitates more informed discussions with patients regarding their treatment options.

Multiple classification systems for both supratentorial and posterior fossa skull base meningiomas have been introduced, utilizing criteria such as location, size, volume, surrounding elements, and encasement of vascular or nervous structures [1,3,4,8,22,24,29]. The diversity of these classification systems complicates the comparison of results across various treatment strategies for these tumors. A major drawback is that tumors with vastly different surgical risks are often classified using similar terminology, leading to significant variation in treatment outcomes and treatment options offered to patients in different medical centers [21].

The presence of an arachnoidal cleavage plane is consistently cited in the neurosurgical literature as one of the most crucial factors predicting the possibility of total tumor removal [30,31]. Given the widely acknowledged importance of the arachnoid and the consistent observation of similar interactions between subarachnoid cisterns and meningiomas, we propose that incorporating the description of this interaction, along with other characteristic patterns, into existing classification systems can enhance our ability to differentiate these pathological entities. This integrated approach could benefit radiologists and neurosurgeons in accurately describing these tumors and planning surgeries. It would aid neurologists in better understanding the future medical needs of these patients, facilitate informed discussions with patients regarding surgical preparation and realistic treatment outcomes, and improve the comparison of various treatment strategies across different medical centers. This variability in treatment outcomes is less common in other medical conditions. With the advent of radiosurgery, endoscopic treatment options, and more advanced surgical tools, comparing these strategies has become increasingly important.

The use of limited vs extensive skull base approaches for skull base meningiomas remains a topic of debate in the neurosurgical community [29,32,33]. The consistent growth patterns of meningiomas in specific locations, particularly in small and medium-sized tumors, suggest that surrounding structures influence their growth, with the arachnoid possibly directing this extension. Like other extra-axial brain tumors, such as craniopharyngiomas, meningiomas may extend along the “path of least resistance” [34]. Vessels and nerves create orifices in the arachnoid membranes as they pass from one cistern to another, and tumors can enlarge these natural passages to develop into adjacent compartments. This explains why, in some cases, the arachnoid membranes separating the tumors from neurovascular elements are partially absent.

During surgery, the space created by the tumor can be utilized, and through progressive debulking, the meningioma can be carefully extracted from neighboring compartments while preserving neurovascular structures. This supports the argument for using smaller, less invasive surgical approaches. A combination of less invasive surgery with radiosurgery could mean less morbidity and similar tumor control to more extensive surgeries, which are not without complications [35].

From our analysis of the connection between subarachnoid cisterns and meningiomas, three key surgical principles emerge:

1. **Guidance for surgical approach based on MRI**
  The visibility of the cisterns surrounding a meningioma on MRI correlates with the ease of dissection and resection. This can guide the selection of the approach of the surgical extent. The use of simple or complex approaches can be decided on a case-by-case basis by carefully inspecting the 'CSF sequences’ on MRI images.
2. **Risk of morbidity with disrupted arachnoid**
  When the arachnoid is absent, or only a single or disrupted arachnoid layer separates the meningioma from neurovascular elements, attempting complete tumor removal carries a high risk of morbidity. Studies have shown that vessel or nerve encasement predisposes patients to higher postoperative deficits, but the presence of an intact arachnoid reduces this risk [2,11,36]. Even when severely compressed, the subarachnoid cisterns provide the necessary, safe space for resection.
3. **Importance of the initial surgical intervention**
  If the goal is total tumor removal, the first surgical intervention is the only opportunity to achieve it. In cases of tumor recurrence, the absence of an arachnoidal plane leads to unpredictable tumor growth patterns and significantly increases the risks associated with tumor removal.

This study is descriptive in nature, and like any descriptive research, it has the inherent limitation of being unable to provide definitive conclusions. This limitation can be mitigated by incorporating multiple observations from diverse groups of researchers and assessing the consistency of findings across studies. The challenge of thoroughly analyzing all connections between arachnoid structures and meningiomas, coupled with a small number of cases, restricts the feasibility of designing comprehensive prospective studies. The retrospective nature of our study is a significant limitation, as it inherently provides results that were collected for purposes other than those of this research. However, the absence of prospective studies is a common challenge in meningioma research, which requires extensive collaboration across various medical specialties, as highlighted in previous studies [21,37]. Given the limited number of cases of skull-base meningiomas, a prospective study in which data needed to precisely define the interactions between arachnoid structures and tumors should probably be a multi-center study. Collecting extensive intraoperative observations made by multiple surgeons, as is needed in a study looking at how the arachnoid influences tumor removal, could be a provocation. As others have noted, the study of skull-base meningiomas has historically relied heavily on individual surgical experience. To establish best practices, this approach needs to evolve towards more standardized, collaborative research efforts [21]. Our study offers a concise overview of the current understanding of the relationship between skull base meningiomas and arachnoid structures.

## CONCLUSION

This paper provides a summarized overview of the current state of knowledge on the interaction between arachnoid structures and skull base meningiomas, which we believe can be useful for all medical specialists caring for people afflicted by this pathology. We emphasize the importance of considering the connection between skull base meningiomas and the surrounding subarachnoid cisterns in surgical planning. We present a conceptual framework illustrating how arachnoid structures affect the growth of skull base meningiomas and their interaction with surrounding neurovascular elements and how this understanding could be integrated into future classification systems.
